# Like Father Like Son: Cultural and Genetic Contributions to Song Inheritance in an Estrildid Finch

**DOI:** 10.3389/fpsyg.2021.654198

**Published:** 2021-06-04

**Authors:** Rebecca N. Lewis, Masayo Soma, Selvino R. de Kort, R. Tucker Gilman

**Affiliations:** ^1^Department of Earth and Environmental Sciences, University of Manchester, Manchester, United Kingdom; ^2^Chester Zoo, Chester, United Kingdom; ^3^Department of Biology, Faculty of Science, Hokkaido University, Hokkaido, Japan; ^4^Department of Natural Sciences, Ecology and Environment Research Centre, Manchester Metropolitan University, Manchester, United Kingdom

**Keywords:** birdsong, song inheritance, vocal learning, cultural evolution, Java sparrow, *Lonchura oryzivora*, song consistency

## Abstract

Social learning of vocalizations is integral to song inheritance in oscine passerines. However, other factors, such as genetic inheritance and the developmental environment, can also influence song phenotype. The relative contributions of these factors can have a strong influence on song evolution and may affect important evolutionary processes such as speciation. However, relative contributions are well-described only for a few species and are likely to vary with taxonomy. Using archived song data, we examined patterns of song inheritance in a domestic population of Java sparrows (*Lonchura oryzivora*), some of which had been cross-fostered. Six-hundred and seventy-six songs from 73 birds were segmented and classified into notes and note subtypes (*N* = 22,972), for which a range of acoustic features were measured. Overall, we found strong evidence for cultural inheritance of song structure and of the acoustic characteristics of notes; sons’ song syntax and note composition were similar to that of their social fathers and were not influenced by genetic relatedness. For vocal consistency of note subtypes, a measure of vocal performance, there was no apparent evidence of social or genetic inheritance, but both age and developmental environment influenced consistency. These findings suggest that high learning fidelity of song material, i.e., song structure and note characteristics, could allow novel variants to be preserved and accumulate over generations, with implications for evolution and conservation. However, differences in vocal performance do not show strong links to cultural inheritance, instead potentially serving as condition dependent signals.

## Introduction

Social learning is an essential component of normal song development for oscine passerines (Beecher and Brenowitz, 2005). In many species, birds that are not exposed to tutor song during the sensitive phases of song ontogeny develop atypical vocalizations, exhibiting unusual note structures, decreased stereotypies, abnormal song length, and other temporal abnormalities (Price, 1979; Marler and Sherman, 1985; Chaiken et al., 1993; Feher et al., 2009; Kagawa et al., 2014). Similarly, birds that are tutored by heterospecifics may incorporate song features of the tutor species in their vocalization, rather than solely producing species-typical songs (Johannessen et al., 2006; Eriksen et al., 2009; Mann et al., 2020). In this way, cultural inheritance of vocal behavior can lead to vocalizations that are shaped by a bird’s social environment (e.g., Greig et al., 2013).

However, a number of other factors also influence song development in juvenile birds. Genetic factors can guide song learning and development. For example, in canaries (*Serinus canaria*), genetic differences show complex interactions with learning and song production, influencing the proportion of low- and high-pitched syllables (Wright et al., 2004; Mundinger, 2010; Mundinger and Lahti, 2014). Genetic factors can also interact with the environment. In Bengalese finches, juveniles produced more accurate imitations of an experimental tutor’s song if songs were played back at a tempo that was similar to their genetic father’s song (Mets and Brainard, 2018, 2019), and the influence of genetic background and environment differed between passively and socially tutored birds (Mets and Brainard, 2018). Other heritable traits, e.g., morphology and neural anatomy, can influence song production. Morphological characteristics, such as body size (Kirschel et al., 2009; Kagawa and Soma, 2013; Derryberry et al., 2018; García and Tubaro, 2018), beak morphology (Podos et al., 2004; Kirschel et al., 2009; Derryberry et al., 2018; García and Tubaro, 2018), and syrinx morphology (Elemans et al., 2015; Christensen et al., 2017), are often correlated with song characteristics. The developmental environment, mediated by social interactions and resource availability, also plays a key role in song development. As song production may incur neural costs during development (Gil and Gahr, 2002), early developmental stress, such as sibling competition (Soma et al., 2006) or nutritional stress (Nowicki et al., 1998), may influence adult songs. Social interactions may guide song production through fraternal inhibition (Tchernichovski and Nottebohm, 1998) and social reinforcement from parents (Carouso-Peck et al., 2020). Finally, both laying order and maternal androgens may contribute to song development (Soma et al., 2009).

Bird song is thought to advertise the relative quality of the singer and to that effect plays an important role in sexual selection (Gil and Gahr, 2002). Songs are multi-faceted signals with learned and unlearned features. Consequently, different aspects of song can reveal different information about the singer’s quality. The aspects of quality these traits convey depend partly on their pattern of inheritance. Learned or environmentally influenced traits may reveal information about developmental environment or learning ability (Nowicki et al., 2002a,b; Boogert et al., 2008; Zann and Cash, 2008), whereas genetically inherited traits may signal “good” genes, which will be inherited regardless of tutor (Hasselquist et al., 1996; Forstmeier et al., 2009). Both types of traits may also inform potential mates of direct benefits, such as adaption to the local environment (Podos and Warren, 2007; Snowberg and Benkman, 2007; Badyaev et al., 2008; Branch and Pravosudov, 2015) or ability to provision offspring (Buchanan and Catchpole, 2000; Halupka and Borowiec, 2006; Bartsch et al., 2015). Of the various song features, significant attention has been paid to three categories: song structure, acoustic characteristics of notes, and song performance measures, which demonstrate complex inheritance patterns and provide a wide range of information about singer’s quality (Table 1).

**Table 1:** Inheritance patterns of common song features.

Song feature	Measurement	Role of social learning	Role of genetic inheritance	Role of developmental environment
Complexity	Repertoire size (number of note types, song types, etc.; Searcy, 1992) Syntactical complexity (note-to-note transitions; Honda and Okanoya, 1999)	Generational overlap in repertoire and note sequences in normal and cross-fostered individuals suggest a learned component (Grant and Grant, 1996; Soma, 2011)	Genetic predisposition for learning certain song components (Wright et al., 2004; Mundinger and Lahti, 2014)	Song learning may incur costs during development, and developmental stress early in life may influence song characteristics and learning (Gil and Gahr, 2002; MacDougall-Shackleton and Spencer, 2012; Schmidt et al., 2014)
Spectral and temporal characteristics	Acoustic characteristics of notes (frequency, duration, amplitude, etc.; Catchpole and Slater, 2008)	Learning of notes may result in replication of acoustic features of tutor (Ritschard and Brumm, 2011). Learned components may reflect local adaptation (Podos and Warren, 2007)	Inherited components may reveal singer quality (e.g., body size and genetics; Forstmeier et al., 2009; Hall et al., 2013)	Stress in early development may influence note production and reduce note copy accuracy (MacDougall-Shackleton and Spencer, 2012)
Performance	Song rate, song amplitude, duration, and trill performance (Cardoso, 2017; Podos and Sung, 2020) Song and/or note consistency (Sakata and Vehrencamp, 2012; Botero and de Kort, 2013)	Complex interaction between tutor learning and individual quality; low quality birds may not be able to reproduce high performance models (Botero and de Kort, 2013); and high quality pupils may increase performance of low quality models (Lahti et al., 2011)	Song performance may correlate with heritable features, e.g., body size (Ballentine, 2009; Kagawa and Soma, 2013) Genes and gene x environment interactions could affect feedback processing and other factors influencing performance (Sakata and Vehrencamp, 2012)	Song production involves coordination of complex motor patterns, high energy requirements and physical constraints and may be more indicative of current condition (Gil and Gahr, 2002; Botero and de Kort, 2011; Schmidt et al., 2014)

Although song inheritance patterns are well-understood for a handful of model species, whether these patterns replicate more broadly across species, and particularly in rapidly diverging lineages and species of conservation concern, is not known. We studied patterns of song inheritance in the Java sparrow (*Lonchura oryzivora*), an estrildid that is endangered in its native range but common in captivity and invasive in some locations (BirdLife International, 2018). We examined an existing song library (Kagawa and Soma, 2013; Ota and Soma, 2014; unpublished data) with songs from multiple generations of father-son pairs for which the genetic pedigree was known. Some birds in our dataset were reared by their genetic fathers, and others were cross-fostered by social fathers with songs that were also included in the dataset. We mined our data for patterns of social learning, genetic heritability, and environmental effects on the development of song structure, note spectral and temporal characteristics, and vocal performance measures, and we quantified evidence for the patterns that we found. Based on previous findings in this and other species, we expect that (1) song structure will be socially inherited, (2) spectral and temporal characteristics of notes will be socially learned but may also have some non-learned components, and (3) vocal performance, measured as vocal consistency, will be genetically inherited.

## Materials and Methods

### Study Population and Recordings

The Java sparrow is an estrildid finch native to South East Asia, and is commonly kept in captivity (Restall, 1996). Males learn to sing a single song type, typically containing 2–8 note types, during a critical period in the nest, with song learning estimated to end at around 150–180 days (Ota and Soma, 2014). As in other estrildids, songs are only used as part of courtship displays (Kagawa and Soma, 2013), which in Java sparrows, also include duet dancing (Soma and Iwama, 2017). Song learning requires social interactions and, as such, juveniles in laboratory settings are most likely to learn from their social fathers if they do not interact with other adult males (Soma, 2011). Inspection of spectrograms suggests that sons produce copies of their fathers’ songs (Soma, 2011), but the relative contributions of cultural and genetic inheritance, and the rearing environment have not been assessed.

**Figure 1:**
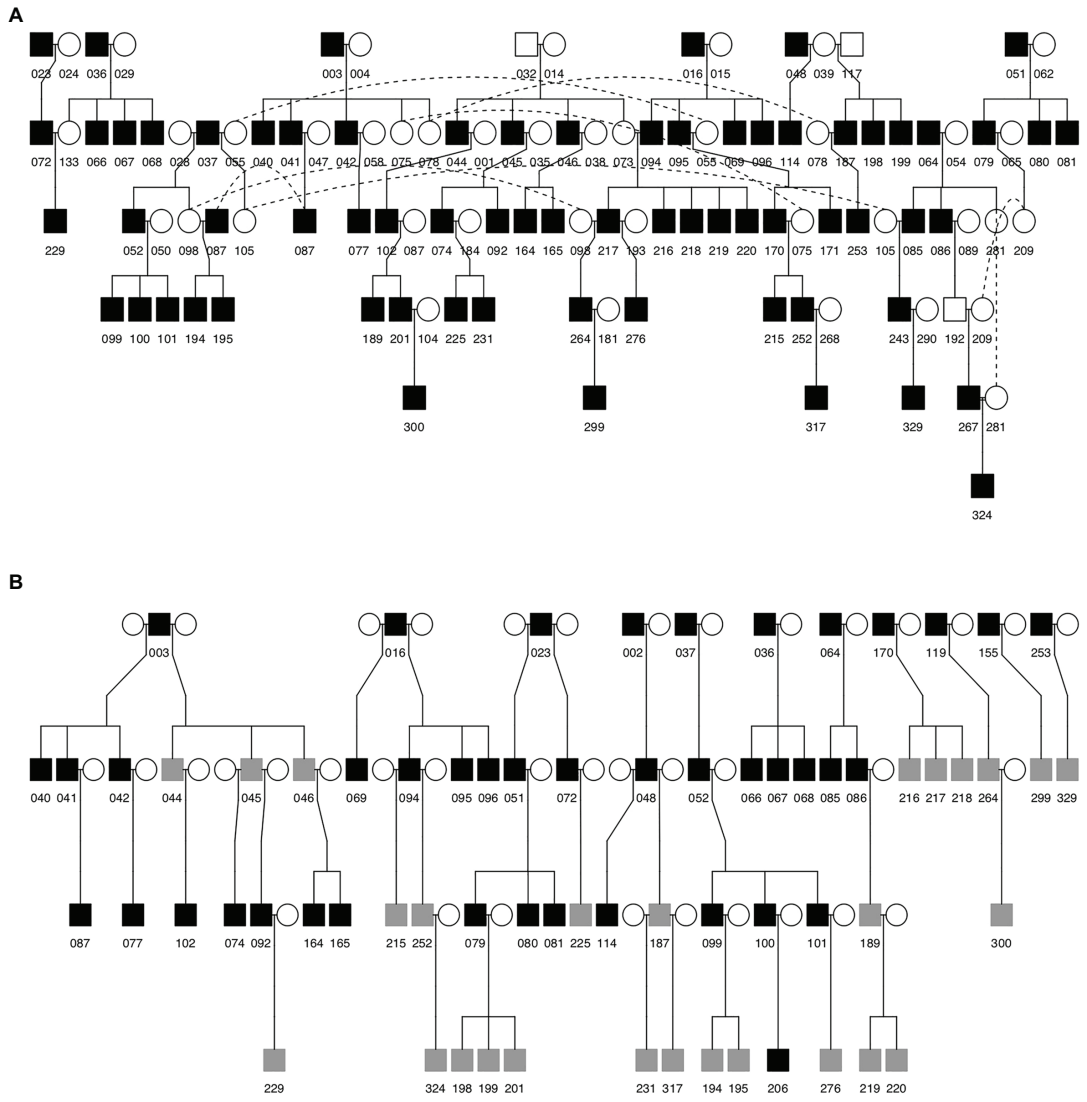
Genetic **(A)** and social **(B)** pedigrees of Java sparrows included in this study. Squares indicate males and circles indicate females. Numbers indicate bird identity. Filled (open) squares indicate that songs for that male were (were not) available for study. Gray squares in the social pedigree indicate individuals that were cross-fostered. Dotted lines in **(A)** connect the same individual where it appears multiple times in the pedigree. In the social pedigree **(B)**, the identities of social mothers are not known. Separate clutches in **(B)** are represented as having different social mothers in the pedigree.

We examined song inheritance in a laboratory population of Java sparrows (Hokkaido Univ.) with a known genetic pedigree (Figure 1A) and known social relationships between males (Figure 1B). Founding individuals were obtained from a range of pet shops and breeders. When breeding, each pair was kept in a separate breeding cage. Nests were inspected regularly, and eggs were cross-fostered when multiple nests with eggs were available. During rearing, each cage was visually, but not audibly, isolated and juveniles remained in the cage with their social parents until they were ~180 days old. This ensures that song learning is from the social father only (Soma, 2011).

Recordings were selected from archival data collected between 2011 and 2020. All recordings were made with 44.1 kHz sampling rate and 16-bit resolution and saved as WAV files. Recordings consist of individual birds singing alone in a soundproof chamber. Recordings were taken using digital sound recorders with built-in microphones, which were placed ~20 cm from the bird’s cage. Several different recorders were used for the archival data collection (Marantz PMD 661, Zoom Q3HD, TASCAM DR-100 MKIII).

### Song Selection

**Figure 2:**
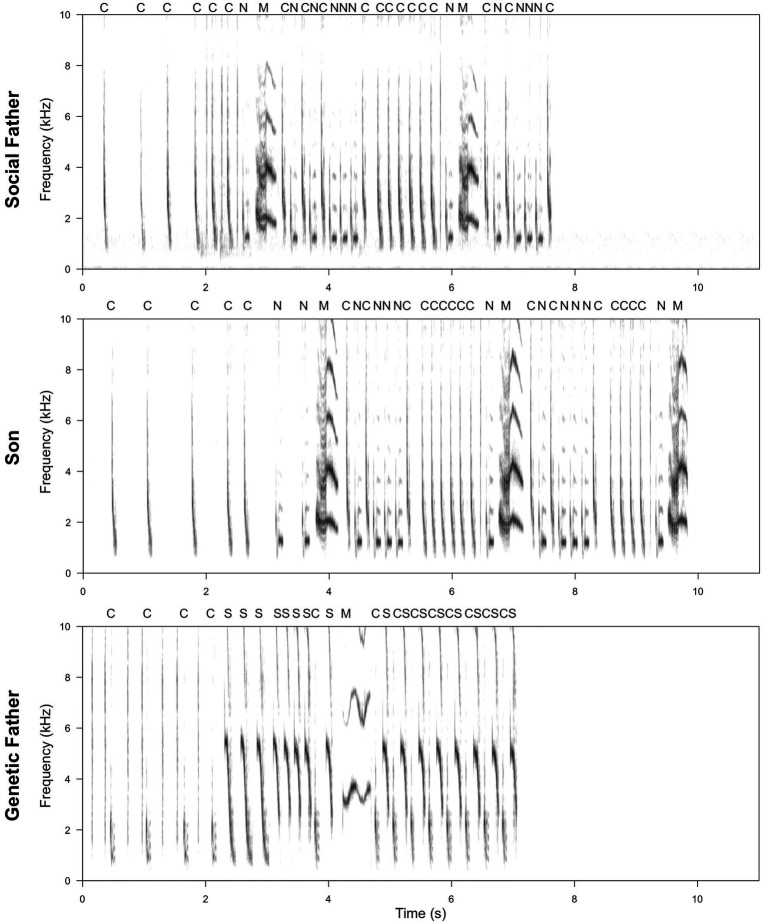
Example of a spectrogram comparison of a son’s song compared to that of his social and genetic father. Letters above the spectrogram represent note types. Spectrograms were produced using SEEWAVE package (Sueur et al., 2008; window length = 512, overlap = 50%). The son produces 100% of the note types in the social father’s song (C, N, and M), including one that is not sung by the genetic father (N). However, one note type produced by the genetic father is not included in the son’s song (S). Transitions between note types in the son’s song are more similar to those in the social, rather than genetic, father’s song, with 86% of social father’s transitions represented, compared to only 29% of genetic father’s transitions. Where note types are present in all three individuals, visual inspection suggests that the acoustic characteristics of notes produced by the son more closely resemble those of the social father (particularly apparent for note type M).

Our dataset included 58 father-son pairs for which the songs of both the son and the social father had been recorded. Of these, 28 sons were raised by their genetic fathers and 30 were raised by social fathers that were not their genetic fathers (see Figure 2 for example songs). Archive data also included a small number of birds that had the opportunity to learn from multiple tutors. These birds were not included as “sons” in the dataset, as we could not ensure that their song learning was confined to the social father. However, birds with multiple tutors exhibited normal adult song and were included in the dataset as fathers if they raised or fathered sons. Altogether, the dataset included 73 birds for which songs had been recorded: the 58 sons identified above and 15 additional birds that had raised (social father) or fathered (genetic father) sons but for which the father’s song was not available. Thirty-one birds entered the dataset as both fathers and sons.

For each bird, we studied songs recorded within a single week. This is important because song features change with age in some species, including other estrildid finches (e.g., Kao and Brainard, 2006; Ballentine, 2009; de Kort et al., 2009; James and Sakata, 2014, 2015, 2019). If a bird was recorded at multiple time points, then recordings from “middle” age (~2–5 years) were preferentially chosen. If multiple recording dates were available within this time frame, then one time point was chosen at random. If at least eight suitable songs were available from the chosen time point, songs from this time point were used in analyses. If a time point with eight or more songs could not be found when a bird was 2–5 years old, then we first chose recordings where the bird was older than 2–5 years, and only chose recordings from 1 to 2 years when older recordings were not available. Recordings in which the bird was over 1 year old were preferred, even if more full songs were available when the bird was younger, since some changes in singing behavior are apparent between song crystallization and 1 year of age (Ota and Soma, 2014). Across all birds, where more than 10 full songs were available from a single time point, 10 songs were randomly selected. If fewer than 10 songs were identified in every time point for a particular bird, all songs from one time point were used. Overall, this resulted in a total of 676 songs from 73 individuals (average of 9.3 songs per individual, range 3–10, only three individuals with <5 songs). The age of birds at recording ranged from 0.41 to 8.83 years, with an average of 3.2 years, and with seven birds recorded at <1 year of age (sons: mean age = 3.1 years, range = 0.41–8.85 years; fathers mean age = 2.58 year., range = 0.41–6.02 years).

### Note Classification and Measurement of Acoustic Characteristics

#### Segmenting Songs Into Notes

Songs were manually segmented into individual notes using the sound analysis software Koe (Fukuzawa et al., 2020; window length = 512, window overlap = 50%, time-axis zoom = 400%, contrast 100%). Mechanical sounds like bill clicks (Soma and Mori, 2015) were excluded from our analyses, and we focused on the production and inheritance of vocal sounds (i.e., notes). A song was defined as a series of notes with inter-note-intervals (gap between notes) of <1 s (Kagawa and Soma, 2013). Manual note selection can introduce measurement error (Zollinger et al., 2012), particularly if recording methods are not consistent. However, the recordings used in this study were taken in controlled conditions with minimal background noise, so the beginnings and ends of individual notes could be easily identified.

#### Manual Note Classification

**Figure 3:**
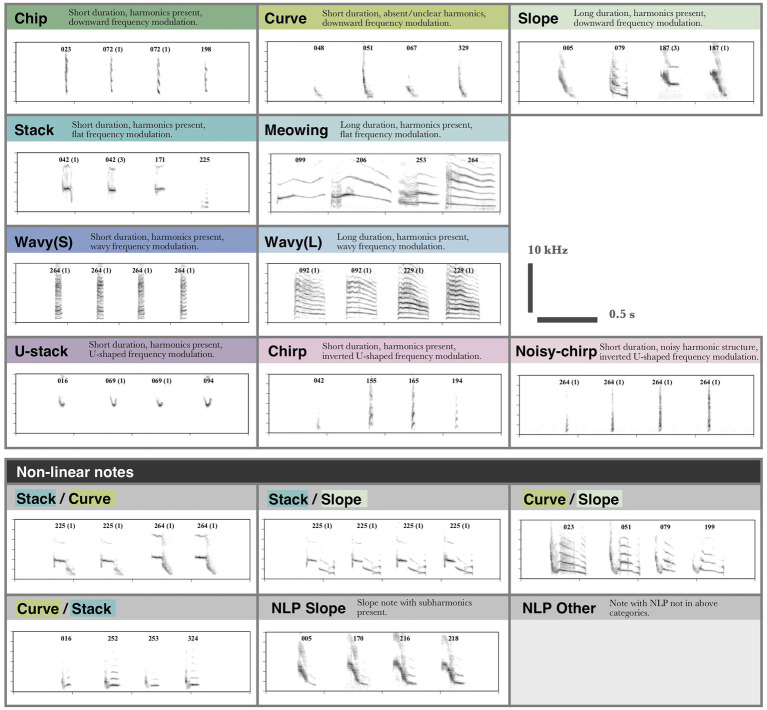
Categories used for note type classification. Note type categories were defined based on frequency modulation, harmonic structure, length, and presence of non-linear phenomena. Notes are labeled to indicate the individual that produced them. Notes produced by different individuals are automatically classed as different subtypes, as subtypes were not aligned between individuals. Where multiple examples from a single individual are shown the subtype is indicated in brackets.

Notes were classified based on a suite of characteristics (e.g., presence of harmonics, frequency modulation, length, and presence of non-linear phenomena), resulting in 16 note types (Figure 3). In total 22,972 notes were segmented and classified (as in Figure 3). A second observer who was naïve to Java sparrow song reclassified a random subset of songs (two songs per individual, 146 songs total, 4,915 notes) to determine the repeatability of manually assigned note types. The second observer was provided with a definition of each note type and eight example notes of each type shown at 100 and 400% time axis zoom. Inter-observer repeatability was high, with agreement of 97.5%.

#### Computational Note Classification

**Figure 4:**
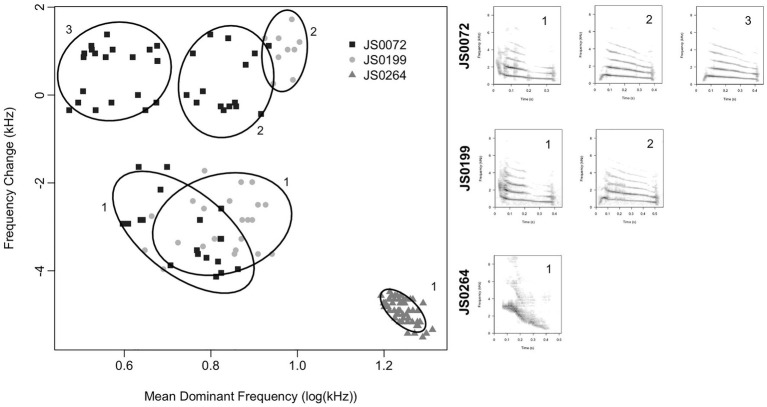
Subtypes observed within a single note type for three representative Java sparrow males from this study. Ellipses show the 80% inclusion space for each cluster. Subtypes are labeled within birds and example spectrograms of each subtype for each bird are included. Subtypes produced by different birds may be distinct or partly overlapping. Thus, it is not clear whether clusters represent different notes, or the same note sung differently. For ease of representation, we show only two note features (mean dominant frequency and frequency change), but patterns are similar for other combinations of features.

The notes belonging to a manually assigned note type and produced by an individual bird may not be monomorphic. Rather, note types may be partitionable into subtypes with distinct acoustic characteristics (Figure 4). These subtypes are broadly comparable with “syllables” in studies of other bird species (Catchpole and Slater, 2008). It is not clear whether note types or subtypes are more biologically relevant, so we studied both in our analyses. We classified notes into subtypes by Gaussian mixture modeling using the R package mclust (Scrucca et al., 2016). We based our classification on a subset of three characteristics – duration, mean dominant frequency, and dominant frequency change. We chose these characteristics because they can be reliably measured even for very short notes (i.e., <70 ms), and we needed to classify all notes in the dataset to analyze song structure. For each set of notes belonging to a particular note type and produced by an individual bird, we fit Gaussian mixture models with up to nine clusters, and chose the optimal number of clusters to minimize the Bayesian Information Criterion of the fitted model. We assigned each note to the cluster to which it was most likely to belong, and we called these clusters “subtypes.” If a bird produced a note type fewer than five times, we assumed that all notes of that type produced by that bird belonged to a single subtype. We did not attempt to equate subtypes produced by different birds. Subtypes produced by different birds may be overlapping, partly overlapping, or may not overlap at all (Figure 4), and, therefore, equating subtypes produced by different birds is not straightforward. Therefore, direct comparisons between note subtypes produced by different birds, e.g., to assess the cultural inheritance of acoustic characteristics at the level of note subtypes, were not possible.

#### Measurement of Spectral and Temporal Characteristics

**Figure 5:**
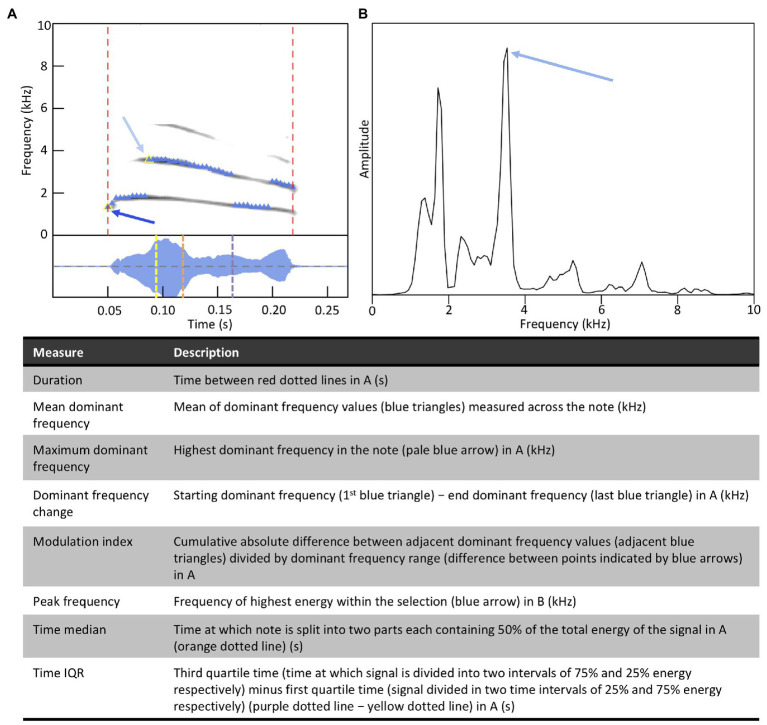
Definitions of acoustic characteristics measured for each note. Panels **A** and **B** support definitions presented in the table.

To measure the acoustic (i.e., spectral and temporal) characteristics of notes, recordings were first high-pass filtered using a FIR filter at 375 Hz to remove low frequency background noise. For each note, we used the specan function (frequency range 0.4–22.05 kHz, window length = 512, overlap = 50%, amplitude threshold = 2%) in the warbleR package (Araya-Salas and Smith-Vidaurre, 2017) in R (version 3.6.3; R Core Team, 2020) to measure acoustic characteristics. Specifically, we measured (i) the mean dominant frequency of the selection, (ii) the dominant frequency change, (iii) the maximum dominant frequency in the selection, (iv) the modulation index, (v) the peak frequency within the selection (based on the mean frequency spectrum), (vi) the note duration, (vii) the time median, and (viii) the time interquartile range (IQR) [Figure 5; see Araya-Salas and Smith-Vidaurre (2017) for further information]. We log transformed the note duration and the spectral characteristics (i.e., mean dominant frequency, maximum dominant frequency, and peak frequency) to homogenize variance. We double log transformed the modulation index, and then set values with no modulation to the smallest detectable modulation in the dataset (i.e., Winsorizing; Tukey, 1962). Double log transformation makes units difficult to interpret. However, because our goal is to regress the acoustic characteristics of sons’ notes on the same acoustic characteristics in the notes of their social fathers, the regression coefficients in our analyses are unitless. We normalized the time median and the time IQR by dividing them by the duration of the notes in which they were measured to obtain values between zero and one. This ensures that the measurement of the energy distribution over time is independent of the note duration.

### Data Analysis

#### Song Structure Analysis

We studied the inheritance of song structure computed at the levels of (i) note types and (ii) note subtypes. We represented each song as a series of note (sub)types, and computed the number of notes, the number of note (sub)types (i.e., repertoire size), the Shannon index, the sequence linearity (Scharff and Nottebohm, 1991), and the first and second-order differential entropies (Schmitt and Herzel, 1997) at each level. One note type was not reliably classified into subtypes by our Gaussian mixture models, and we assigned all instances of this note type to a single subtype for analyses. For each bird, we took the mean of each song structure measure across all songs in the dataset to obtain a single phenotype per bird and per measure. We regressed the sons’ phenotypes on the phenotypes of the social fathers. A significant positive regression coefficient indicates that the phenotype is culturally inherited. In particular, coefficients close to one indicate little regression to the population mean, so the characteristics of a song lineage are likely to persist for many generations, and regression coefficients close to zero indicate that the characteristics of a song lineage rapidly decay toward the population mean. To test for genetic heritability, we included a random effect of relatedness in the regression, where the relatedness matrix was computed from the known pedigree of birds in the dataset. Including the full relatedness matrix rather than just the genetic father in our analysis allows us to take advantage of information about more distantly related individuals, and increases our ability to detect genetic effects. This is particularly important because some birds were raised socially by their genetic fathers, which makes it difficult to disentangle social learning from genetic inheritance without considering similarities among more distant relatives. A significant effect of relatedness would indicate that, even when controlling for potential learning from the social father, birds’ song phenotypes were more similar to those of related than those of unrelated individuals. We included a random effect of the clutch ID to account for similarities among nestmates that are not due to learning from the social father. This could be due to factors including, but not limited to, common rearing conditions, differences in the quality of parental care, the sizes of broods, and the identity of social mothers [whose song preference and social feedback may influence the song learning and production of her sons (Carouso-Peck and Goldstein, 2019; Carouso-Peck et al., 2020)]. Because Java sparrow songs can change with age following crystallization (Ota and Soma, 2014), we included a fixed effect of log-transformed age in the model. We removed the effect of age from the model if it not at least marginally significant (i.e., *p* > 0.1). In this and subsequent analyses, we log-transformed the song phenotypes and refit the models if necessary to homogenize variance in the residuals. We fit models using the lmekin function in the R package coxme (Therneau, 2018), and we tested the significance of random effects using likelihood ratio tests. Likelihood ratio tests of random effects are known to be conservative (Pinheiro and Bates, 2000).

#### Analysis of Acoustic Characteristics

Next, we asked whether the acoustic characteristics of sons’ notes were similar to those of their social fathers when they produced the same note types. We computed the mean value for each characteristic of each note type as produced by each bird. If a bird did not produce a particular note type at least five times, then we excluded that note type from the analysis for that bird. Thus, if an individual bird produced four different note types at least five times each, then we computed four means for that bird. The variances of acoustic characteristic values for the note types in our dataset differed by up to three orders of magnitude. We z-scored acoustic characteristic values within note types to homogenize variance, as homogeneity of variance is a fundamental assumption of our regression models. Finally, for each acoustic feature, we regressed the sons’ mean for each note type on the social fathers’ mean for the same note type. If a social father produced notes of a particular type but his son did not, or vice versa, then that note type did not appear in the analysis for that social father-son pair. A significant positive relationship between the social fathers’ mean acoustic characteristic value and the sons’ mean acoustic characteristic value indicates that sons learned how to produce individual note types from their social fathers. We included fixed effects of note type and log-transformed age in the model, and we included random effects of the relatedness matrix, the clutch ID, and the individual ID of the son. Including a fixed effect of note type accounts for the fact that different note types have different mean characteristic values, and prevents us from inferring that fathers and sons produce notes with similar characteristics simply because they produce the same note types. The effect of age accounts for the possibility that older birds produce notes differently than younger birds. We removed this effect from the model if it was not at least marginally significant (i.e., *p* > 0.1). The random effect of the relatedness matrix controls for potential heritability of acoustic characteristic values, and the effects of clutch and individual control for correlations among sons’ acoustic characteristic values that are not due to learning from their social fathers.

#### Performance Analysis

The ability to produce individual note types consistently is thought to be a signal of mate quality in a number of species, and birds are likely to compare notes that are produced within the same song (Sakata and Vehrencamp, 2012; Botero and de Kort, 2013). Therefore, we wanted to know whether the ability to produce note types consistently within a song is culturally transmitted, genetically heritable, and/or influenced by the rearing environment. We cannot study consistency at the level of note types. Some birds produce multiple note subtypes within note types. If sons learn which subtypes to produce from their social fathers, as our results suggest they do, then studying consistency at the level of note types will confound the learning of note type consistency with the learning of note subtype. Therefore, we studied consistency at the level of note subtypes. To achieve this, we (i) assessed the within-song consistency of each note subtype produced by each bird, (ii) standardized across note subtypes to control for the fact that some note subtypes may be more difficult to produce consistently than others, (iii) computed the mean consistency for each bird across all note subtypes that the bird produced, and (iv) regressed the consistency of sons on the consistency of their social fathers.

We assessed the consistency of note subtypes in three ways: by comparing (i) the variance of individual acoustic characteristics, (ii) the dynamic time warping distance, and (iii) the spectral cross correlation among notes. For the variance measures and dynamic time warping distance, lower values indicate greater consistency, and for spectral cross correlation, higher values indicate greater consistency. For each song produced by each bird, we calculated the variance of the acoustic characteristics of each note subtype that appeared in that song more than once. We examined the same acoustic characteristics that we used previously to classify notes to subtypes (i.e., the logarithms of duration and mean dominant frequency, and change in dominant frequency over the course of the note). Within each song produced by each bird, we measured the mean squared pairwise dynamic time warping distance and the median pairwise cross correlation between notes of the same subtype. At the assessed window length (512), warbleR does not accurately measure the change in dominant frequency for notes less than 20 ms in duration, so for change in dominant frequency, we excluded these notes from the analysis. We excluded the note type that was not reliably classified into subtypes by our Gaussian mixture models from all consistency computations, due to the need to accurately identify subtypes in this analysis.

For each bird, we took the weighted average (or, for spectral cross correlation, the weighted median) across all songs produced by that bird, with each song weighted according to the number of times the note subtype appeared. This produced a value for each consistency measure for each note subtype produced by each bird across all songs that the bird produced. We cannot accurately estimate the variability of a note subtype within songs unless that note subtype is frequently repeated within the same song. Thus, we excluded note subtypes for individual birds if the total number of times the bird produced the note subtype was not greater by at least five than the total number of songs in which the bird produced the note subtype. So, if a bird produced a note subtype in five songs, then the subtype would be included in the analysis if it were produced a minimum of 10 times. For the acoustic characteristic variances and the dynamic time warping distance, we log transformed the values to normalize error. For duration, two birds produced one note subtype (out of 420 birds by note subtype combinations in the data) with variabilities more than 9 SDs below the population mean. These are likely to be errors due to the fact that warbleR measures the durations of notes in discrete units. Therefore, we Winsorized these two values to the smallest observed variability among the other bird by note type combinations in the data.

At this point in the analysis, we had obtained a single value for each of our consistency measures for each note subtype as produced by each bird. However, some note subtypes may be more difficult to produce consistently than others, and individual birds produce different note subtypes. So, to make comparisons among birds, we needed to standardize consistency measures across note subtypes. In our analysis of acoustic characteristics, we standardized across note types produced by different birds by mean centering on each note type. We could do this because note types produced by different birds can be clearly equated. However, note subtypes produced by different birds cannot be clearly equated, so we cannot mean center at the level of note subtypes. Therefore, we used a modeling approach to control for differences in consistency among note subtypes. We assumed that, within each note type, the consistency of note subtypes might depend on the acoustic characteristic values of the subtype and on the number of times the bird produced the subtype (e.g., if birds learn to produce notes consistently by practicing them more often). For each subtype produced by each bird, we computed the means of the log transformed duration, log transformed mean dominant frequency, and frequency change, and we counted the number of times the bird produced the subtype and the number of songs in which the bird produced the subtype. We fit our observed consistency values to mixed linear regressions that included fixed effects of every combination of these five predictors, as well as the second and third-order interactions among the acoustic characteristics, and included the note type as a categorical variable. To avoid attributing any effect of individual birds to these predictors (and thus overfitting due to pseudoreplication), we included random effects of the individual bird and the individual bird’s natal clutch in the model. We fit the models by maximum likelihood using the R package lme4 (Bates et al., 2015). Fitting by maximum likelihood allows us to weight each model according to its Bayesian Information Criterion (Pinheiro and Bates, 2000). Then, we computed the model-weighted regression coefficients for each of the predictors we considered in the full model (Burnham and Anderson, 2002). Finally, we corrected the observed consistency value for each note in the dataset by subtracting the model-weighted fixed effects of its predictors. This left us with a set of residuals that are measures of consistency with the effects of note subtype removed (i.e., with an expected value of zero for every note subtype), but with any random effects of clutch and individual still included in the measure. We computed a single value for each consistency measure for each bird by averaging across all note subtypes that the bird produced.

To ask whether sons learn their consistencies from their social fathers, we regressed the sons’ residual consistencies on the residual consistencies of their social fathers for the same consistency measures. We included the sons’ log-transformed age in the model, because in many species, birds produce notes more consistently as they get older (Kao and Brainard, 2006; Botero et al., 2009; de Kort et al., 2009; Rivera-Gutierrez et al., 2010; James and Sakata, 2019). We included a random effect of the relatedness matrix in the model to account for potential genetic heritability of consistency, and we included a random effect of natal clutch in the model to account for effects of the rearing environment. If sons learn their consistencies from their social fathers, and if consistency changes with age, then sons are most likely to learn from the consistencies that their social fathers displayed at the time of rearing. In general, the songs of social fathers in our data were not recorded at the time of rearing (mean age at recording 2.64 years, sd 1.47 years; mean age at rearing of sons 1.87 years, sd 1.03 years; mean difference 0.76 years, sd 1.70 years). Therefore, we needed to correct social fathers’ consistency measures to reflect their age at time when they were rearing sons. We could not do this by simply including the difference in the fathers’ log-transformed ages at the times of recording and rearing sons in the model as a predictor. This would add a free parameter to the model, but in practice, the necessary correction for the fathers’ consistency is fully determined by the difference in his ages at the times of recording and rearing sons and by the coefficient of log-transformed age in the fitted model. Therefore, we started by fitting models using the social father’s uncorrected consistency as a predictor. Then, we corrected the fathers’ consistencies using the coefficient of log-transformed age in the model we had just fit, and we refit the model. We repeated this process until the effect of age in the fitted model and the effect of age used in the correction differed by less than a proportion of 10^−6^ of the fitted value. This resulted in models with the fathers’ consistency corrected according to the fitted coefficients of the model itself. If the effect of age was not at least marginally significant (i.e., *p* > 0.1), we removed log-transformed age from the model and refit, without correcting the fathers’ consistency phenotype. We left the social fathers’ residual consistency phenotype in the model even if it was not statistically significant. This ensures that we do not incorrectly attribute an effect of learning from the social father to other aspects of the rearing environment simply because the effects of learning are too small to detect with confidence. If there is no learning from the social father, then including the social fathers’ phenotypes in the model will incorrectly attribute some clutch effects to learning from the social father, and so reduce the apparent effect of clutch. We assessed the significance of random effects (i.e., relatedness and clutch) using likelihood ratio tests. Likelihood ratio tests for random effects are known to be conservative (Pinheiro et al., 2017).

## Results

**Figure 6:**
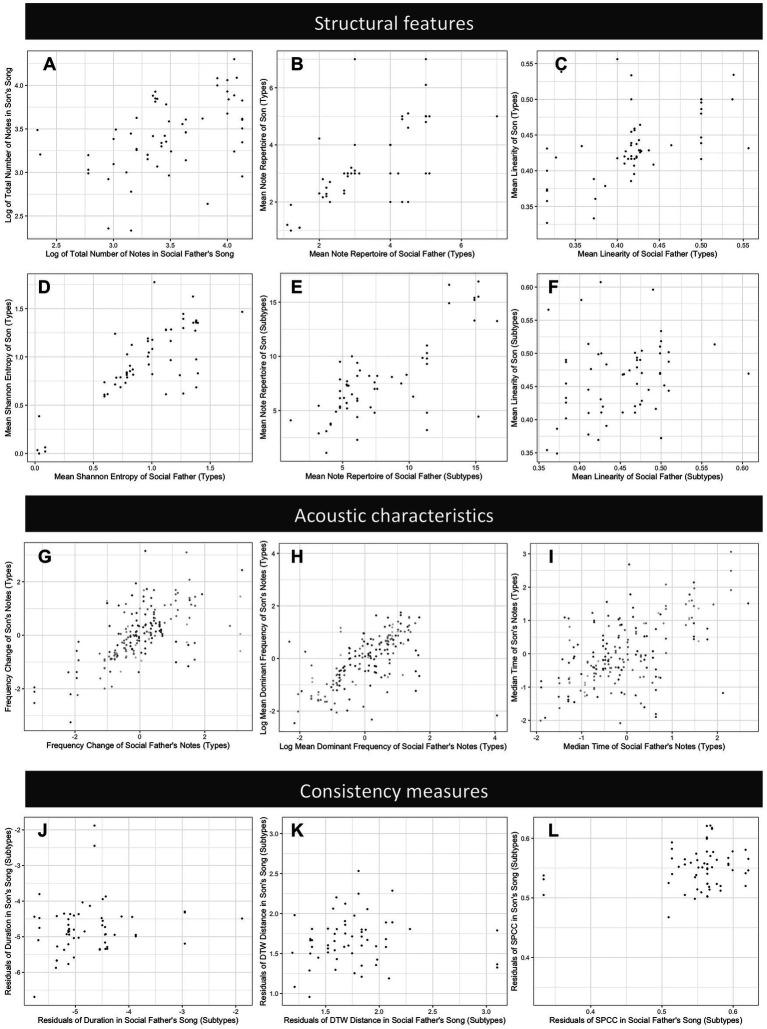
Comparison of song features across songs produced by sons and their social fathers. Plots represent a subset of features examined and show typical patterns for each set of features. Plots **(A–F)** compare structural features: **(A)** mean total number of notes in song (song length), **(B)** mean note type repertoire (manually assigned note types), **(C)** mean song linearity (manually assigned note types), **(D)** Shannon entropy (manually assigned note types), **(E)** mean note subtype repertoire (computationally assigned note subtypes), and **(F)** mean song linearity (computationally assigned note subtypes); **(G–I)** compare acoustic characteristics of note types (z-scored), with shading representing different note types: **(G)** frequency change, **(H)** mean dominant frequency, and **(I)** time median; and **(J–L)** compare measures of vocal consistency of note subtypes: **(J)** variance of mean dominant frequency, **(K)** dynamic time warping distance, and **(L)** spectral cross correlation.

For the whole song analyses, we found strong evidence that features of song structure are socially learned; son’s songs were similar to those of their social father (Table 2). For manually assigned note types, there was a strong positive relationship between the social father’s and son’s songs for all measures with a large associated effect size (all *p* < 0.001, Table 2; Figure 6), i.e., for the structural features measured, sons produced songs with features closely resembling those of their social father. Song structure at the level of computer-assigned note subtypes was also learned (repertoire size, *p* < 0.0001; Shannon diversity, *p* < 0.0001; first order differential entropy, *p* = 0.0014; song linearity, *p* = 0.0027; Figure 6), but the effect sizes were generally smaller than those reported for manually-assigned note types. There was no strong evidence that age of the bird at time of recording influenced any structural measure, although positive relationships were found for the number of notes (*p* = 0.068), the second order differential entropy (*p* = 0.056) when considering manually assigned note types, and the first order differential entropy (*p* = 0.024) when considering note subtypes. There was no evidence that genes or the rearing environment influenced song structure (Table 2). In this analysis, we treated the one note type that was not reliably clustered into subtypes as if it were a single subtype, but our results are qualitatively unchanged if we conduct the same analysis using the original computationally assigned subtypes (Supplementary Information 1).

**Table 2:** Results of mixed-effect models for structural features of songs^†^.

Response	Social father’s phenotype	log(age)	Relatedness	Clutch	Relatedness or clutch
Number of notes^*^	0.45 *p* = 0.0002	0.12 *p* = 0.068	*p* = 0.11	*p* = 0.15	*p* = 0.10
Note types (manually assigned)					
Repertoire size^*^	0.82 *p* < 0.0001	0.007 *p* = 0.88	*p* = 0.99	*p* = 0.98	*p* > 0.99
Shannon entropy	0.80 *p* < 0.0001	−0.006 *p* = 0.88	*p* = 0.98	*p* = 0.98	*p* > 0.99
Song linearity	0.39 *p* = 0.0006	0.0046 *p* = 0.54	*p* = 0.89	*p* = 0.10	*p* = 0.27
1st order entropy	0.81 *p* < 0.0001	0.012 *p* = 0.51	*p* = 0.71	*p* = 0.79	*p* = 0.71
2nd order entropy	0.70 *p* < 0.0001	0.035 *p* = 0.056	*p* = 0.97	*p* = 0.94	*p* > 0.99
Note subtypes (computationally assigned)					
Repertoire size^*^	0.51 *p* < 0.0001	0.095 *p* = 0.14	*p* > 0.99	*p* = 0.17	*p* = 0.40
Shannon entropy	0.50 *p* < 0.0001	0.086 *p* = 0.13	*p* > 0.99	*p* = 0.33	*p* = 0.62
Song linearity	0.37 *p* = 0.0027	−0.015 *p* = 0.11	*p* = 0.99	*p* = 0.98	*p* > 0.99
1st order entropy	0.30 *p* = 0.014	0.060 *p* = 0.024	*p* = 0.83	*p* > 0.99	*p* = 0.98
2nd order entropy	0.13 *p* = 0.33	0.028 *p* = 0.15	*p* > 0.99	*p* = 0.98	*p* > 0.99

* Indicates that response variable was log-transformed.

† Across all birds, structural features were computed from a total of 676 songs with a total of 22,972 notes. For each structural feature, we studied data on 58 social father-son pairs.

For the individual note analyses, we found strong evidence that acoustic characteristics of note types are learned. For all measures considered, there was a strong positive relationship between the notes produced by social fathers and those of sons (all *p* < 0.001), with large effect sizes (Table 3; Figure 6); within categories, sons produced notes that were similar to those of their social father. There was no evidence for a relationship between age at time of recording and any of the acoustic characteristics considered (all *p* > 0.1). There was evidence for an effect of clutch for time median (*p* = 0.014), time IQR (*p* = 0.0070), and mean dominant frequency (*p* = 0.041), indicating that birds from the same clutch were more similar than expected by chance alone (Table 3). We found no evidence for a genetic effect on any acoustic characteristic (Table 3).

**Table 3:** Results of mixed-effect models for acoustic characteristics of notes within songs^†^.

Response	Social father’s phenotype	log(age)	Relatedness	Clutch	Relatedness or clutch
Duration^*^	0.63 *p* < 0.0001	0.040 *p* = 0.67	*p* > 0.99	*p* = 0.99	*p* = 0.86
Time median	0.61 *p* < 0.0001	−0.041 *p* = 0.67	*p* > 0.99	*p* = 0.014	*p* = 0.043
Time IQR	0.60 *p* < 0.0001	−0.080 *p* = 0.43	*p* > 0.99	*p* = 0.0070	*p* = 0.0028
Mean dominant frequency^*^	0.63 *p* < 0.0001	0.095 *p* = 0.27	*p* = 0.96	*p* = 0.041	*p* = 0.12
Maximum dominant frequency^*^	0.68 *p* < 0.0001	0.030 *p* = 0.67	*p* = 0.94	*p* = 0.97	*p* > 0.99
Modularity index^**^	0.53 *p* < 0.0001	−0.13 *p* = 0.18	*p* = 0.82	*p* > 0.99	*p* = 0.98
Frequency change	0.64 *p* < 0.0001	0.12 *p* = 0.13	*p* > 0.99	*p* = 0.54	*p* = 0.83
Peak frequency^*^	0.62 *p* < 0.0001	0.083 *p* = 0.29	*p* > 0.99	*p* = 0.27	*p* = 0.54

* Indicates that response variable was log transformed.

** Indicates that response variable was double log transformed.

† Acoustic characteristics were computed from a total of 20,764 notes, where the note types were produced at least five times by both sons and their social fathers. For each spectral feature, we studied data on 182 social father-son pair x note type combinations.

We found no clear evidence for social learning of vocal consistency (Table 4; Figure 6), but we found a trend suggesting that social fathers with more consistent note durations had sons with more consistent note durations (*p* = 0.094). Older birds produced note subtypes with more consistent durations than younger birds (*p* = 0.0022). However, there was a trend in the opposite direction for spectral cross correlation – older birds appeared to produce less consistent note subtypes (*p* = 0.066). We found no evidence for an effect of age for any other consistency measure (Table 4). There was evidence that the random effects influenced consistency measures in all cases except the variance of frequency change (Table 4). For the variance of mean dominant frequency (*p* = 0.0033), the dynamic time warping distance (*p* = 0.0025), and spectral cross correlation (*p* = 0.015), birds from the same clutch were more similar than we would expect by chance alone. For the variance of duration, there was a random effect of either clutch or relatedness (*p* = 0.0056). However, because birds from the same clutch were always genetic brothers in our data, natal clutch and genetic relatedness are correlated, and we cannot determine which of these explains the effect. Visual inspection of the scatterplots (Figure 6) revealed three potentially influential points, which were all birds from a single clutch. Results remain qualitatively unchanged if we conduct the same analyses excluding these individuals (Supplementary Information 2).

**Table 4:** Results of mixed-effect models for vocal consistency of note subtypes^†^.

Response	Social father’s phenotype	log(age)	Relatedness	Clutch	Relatedness or clutch
Note duration^*^	0.19 *p* = 0.094	−0.34 *p* = 0.0022	*p* = 0.14	*p* = 0.078	*p* = 0.0056
Mean dominant frequency^*^	0.15 *p* = 0.26	−0.16 *p* = 0.19	*p* > 0.99	*p* = 0.0033	*p* = 0.013
Frequency change	0.098 *p* = 0.51	−0.032 *p* = 0.82	*p* > 0.99	*p* = 0.33	*p* = 0.62
Dynamic time warping	0.059 *p* = 0.64	−0.064 *p* = 0.20	*p* > 0.99	*p* = 0.0025	*p* = 0.010
Spectral cross correlation (median)	0.11 *p* = 0.25	−0.010 *p* = 0.066	*p* > 0.99	*p* = 0.015	*p* = 0.028

† Vocal consistency was computed from a total of 18,985 (note duration, mean dominant frequency); 17,917 (frequency change); or 17,808 (dynamic time warping, spectral cross correlation) notes where the same note subtype appeared multiple times in the same song. For each vocal consistency measure, we studied data on 58 social father-son pairs.

* Inspection of scatterplots revealed three potentially influential points, which were all birds from a single clutch. Data were reanalyzed with these three birds removed, resulting in changes in significance of some values (see Supplementary Information 2), but do not change the interpretation of our results.

## Discussion

We examined the roles of cultural and genetic inheritance in shaping song phenotypes. There was strong evidence for the social learning of song structure and the acoustic characteristics of notes. Sons’ song structure and note characteristics were similar to those of their social fathers. There was no effect of genetics, and with few exceptions, no effect of age. Here, and throughout, the effect of age may be limited as analyses were confined to the songs of adult birds, where age-related song changes may be slow to manifest or small in magnitude (James and Sakata, 2014). For some features, there was an effect of the clutch in which the bird was reared, with individuals from the same clutch more similar than expected by chance alone, indicating an influence of the developmental environment. For vocal consistency, we found no strong evidence of social learning or genetic heritability. However, vocal consistency was influenced by the age of the bird and by the clutch in which the bird was reared, again indicating an influence of the developmental environment. Further empirical work will be required to confirm these patterns in Java sparrows and other species as our analyses were largely exploratory.

Sons resembled their social father in all measures of song structure, with no effect of genetic relatedness in any case, suggesting that these traits are culturally inherited within populations. Note repertoire size was similar in sons and their social fathers, as is the case for many other species (Grant and Grant, 1996; Takahasi and Okanoya, 2010; Soma, 2011; Labra and Lampe, 2018). We also found evidence for cultural inheritance of song complexity, as measured by linearity (Scharff and Nottebohm, 1991), and of higher order note sequencing, as measured by differential entropy. Similar sequence learning has been reported recently in Bengalese finch, and birds were more likely to learn note transitions commonly used by their social fathers (James et al., 2020), although we did not examine this in our dataset.

Regression coefficients for the social learning of fathers’ song structure were large, suggesting that there is limited regression toward the population mean in each generation. Thus, sons produced faithful copies of their social fathers’ songs, and differences among song lineages could persist for many generations. When considering note subtypes (i.e., those that were computationally assigned based on clustering of acoustic characteristics), the magnitude of the regression coefficients was smaller. This suggests that regression toward the population mean (as indicated by coefficients closer to zero) is greater when considering note subtypes, and that innovation may be more likely to involve changes among note subtypes than among note types.

Within note types, sons sang notes with similar acoustic characteristics to those of their social fathers. This may mean that birds learn how to produce notes of a particular type from their fathers, i.e., they learn the acoustic characteristic values of their social father’s note types, or it may mean that they learn which distinct note subtypes to produce from their fathers, which would be reflected in similar mean and variance of note type acoustic characteristics in social father-son pairs.

We found no evidence of genetic inheritance of acoustic characteristics; there was no effect of relatedness on any characteristic measured. This is in contrast to findings from a number of other species [e.g., zebra finch (Forstmeier et al., 2009) and Bengalese finch (Kagawa et al., 2014; Mets and Brainard, 2018, 2019)] where genetic differences underpin some differences in acoustic characteristics. It is possible that levels of genetic variation within the present laboratory population were not large enough to assess the genetic heritability of acoustic characteristics. Reduced genetic variability in laboratory compared to wild populations has been reported in other species (Forstmeier et al., 2007). In Bengalese finches and white-rumped munia, strain-specific differences in acoustic characteristics of notes are apparent (Kagawa et al., 2014). However, the two strains have high levels of disparity in morphology (Soma, 2005) and presumably genetics. A potential caveat of our analysis method (using a relatedness matrix) in determining heritability is that relatedness of founders in our population is unknown. As such, individuals may have been more closely related than suggested by our relatedness matrix.

In contrast to song structure and note acoustic characteristics, we found no strong evidence for the cultural inheritance of vocal consistency, which is a common measure of song performance (but see Supplementary Information 2). The vocal consistency of the social father did not predict his sons’ vocal consistency. However, contrary to our predictions, we also found no relationship between genetic relatedness and measures of vocal consistency; birds that were related did not have similar levels of vocal consistency. Although evidence was limited, some interesting patterns are apparent. In all models, effect of social father’s phenotype was positive, which is consistent with social learning. However, the effect sizes are small in comparison to those for song structure and the acoustic characteristics of notes, so, even if such effects exist, we expect that they will be small. There was some evidence that vocal consistency was related to age at recording; older birds had more consistent note duration across vocalizations than younger birds, although this was not the case for other measures of consistency. Increased vocal consistency with age has been reported in a number of studies across a broad range of bird species, for example (Kao and Brainard, 2006; Botero et al., 2009; de Kort et al., 2009; Rivera-Gutierrez et al., 2010). Improved vocal consistency with age may reflect greater opportunity to practice motor patterns involved in vocalizations (Sakata and Vehrencamp, 2012; Botero and de Kort, 2013). Thus, if age indicates good genes because the bird has survived or good parental care because the bird has experience, then vocal consistency may be an honest signal of quality in Java sparrows. However, there was no effect of age on the consistency of mean dominant frequency or frequency change for note subtypes within songs, and no effect of age on the dynamic time warping distance among notes of the same subtype. Similarly, James and Sakata (2014) also found no significant age-dependent changes in the mean or variability in a range of frequency-based syllable features in Bengalese finches, although this also included syllable duration. When considering spectral cross correlation within note subtypes, we report a trend in the opposite direction; older birds tended to sing less consistently than younger birds. Variance of note duration and distance by spectral cross correlation are negatively correlated, so it would be somewhat surprising for either of these patterns to appear by chance alone in the presence of the other. It is, therefore, not clear how vocal consistency relates to age in male Java sparrows. Our findings suggest the need for further examination of vocal consistency in this species, and for including age in analyses when considering similar questions in other species. There is evidence that differences in consistency are salient (de Kort et al., 2009) and can influence female preference (Woolley and Doupe, 2008), male reproductive success (Byers, 2007; Cramer et al., 2011), and social status (Botero et al., 2009) in some species. The consistency measures we studied carry information about a singer’s age and rearing environment (discussed below). However, determining whether these measures are salient to Java sparrows will require further empirical work.

For a range of measurements across the features examined, we found evidence for an effect of clutch. This reflects an impact of the social father or developmental environment independent of the social father’s song. This was especially relevant to vocal consistency, where the majority of measures indicated an important role for the rearing environment. Due to the nature of our dataset, it was not possible to disentangle a number of possible effects, as many factors might contribute to the clutch variable. One possibility is that clutch effects result from differences in the early developmental environment between nests (Nowicki et al., 2002b; Holveck et al., 2008). In the Bengalese finch, birds from larger, male-biased broods had lower song complexity than those from smaller broods (Soma et al., 2006). In this case song features, such as consistency, may be honest indicators of male quality. Vocal development incurs neural costs during early development (Gil and Gahr, 2002) when birds are likely to be exposed to stressors. High quality songs may, therefore, indicate lower stress levels during development (Nowicki et al., 1998; Nowicki and Searcy, 2004). As well as increasing developmental stress, large, male-biased broods may also result in fraternal inhibition of song learning. In zebra finches, birds with more male siblings had shorter motif durations and reduced note numbers compared to their tutors (Tchernichovski and Nottebohm, 1998). Social reinforcement of learning from parent birds may also play a role in clutch-specific differences in learning accuracy, as parents are likely to show variation in the levels of reinforcement provided. In zebra finches, social feedback from both the father and mother was correlated with song learning, with birds that received appropriate input producing more faithful copies of fathers’ songs (Carouso-Peck et al., 2020).

The strong influence of vocal learning, particularly of song structure and the acoustic characteristics of notes, has implications for the evolution and maintenance of song in the Java sparrow. Sons do not precisely copy their social fathers’ songs; there are differences, particularly when considering note subtypes, which may relate to improvisation or copying errors during learning. However, we report large effects of the social father’s phenotype, suggesting that learning fidelity is high for these traits. In this case, novel variants that arise during the learning process may be preserved and accumulate over generations, contributing to population divergence in song and the formation of vocal dialects (Baker and Cunningham, 1985; Catchpole and Slater, 2008). Differences in songs among populations, if coupled with female preference for local song types, can result in pre-mating reproductive isolation and, in some cases, speciation (Kirkpatrick, 2000; Verzijden et al., 2012). The potential role of song in speciation may be of particular interest in Estrildids, as the family has recently undergone a period of rapid speciation (Olsson and Alström, 2020). In a closely related species, the Bengalese finch, female preference for song complexity, coupled with release from selection pressures, has been highlighted as a driver for increasingly complex songs in captivity compared to ancestral wild populations (Okanoya, 2012; Suzuki et al., 2014). While the Java sparrow and Bengalese finch share similar life histories and domestication history, little is known about female preference for song features in Java sparrows. It is, therefore, not possible to predict how female mate choice could impact song evolution in the Java sparrow.

The Java sparrow has been widely bred in captivity as part of *ex situ* conservation efforts (BirdLife International, 2018) and as a popular species in aviculture (Restall, 1996). Genetic and behavioral change is frequently reported in captive breeding programs and can accumulate over relatively short time periods, spanning few generations (Håkansson and Jensen, 2005; Frankham, 2008; Suzuki et al., 2013), and these differences may extend to vocal behavior (Tanimoto et al., 2017). The potential for song evolution and cultural divergence in the Java sparrow is, therefore, likely to be of interest to conservation practitioners, as vocal changes may influence the success of conservation programs and in particular reintroduction programs (Lewis et al., 2020; Crates et al., 2021).

Overall, we find that cultural processes play a large role in the song inheritance of Java sparrow, influencing song structure and complexity, as well as acoustic characteristics of notes, in line with findings in other species. Social inheritance of these features has the potential to influence the formation and maintenance of population specific differences, with implications for evolution and conservation in Java sparrows. However, we found no clear evidence for the inheritance of a performance-related factor, vocal consistency, which was instead related to age at recording and clutch-specific differences. While we did not find a relationship with genetic relatedness, our findings support the hypothesis that vocal consistency is an honest signal of quality, revealing information about the age and developmental environment of the signaler.

## Data Availability Statement

The original contributions presented in the study are publicly available. This data can be found here: FigShare; https://doi.org/10.48420/14555247.

## Ethics Statement

The animal study was reviewed and approved by The University of Manchester Animal Welfare and Ethics Review Board.

## Author Contributions

RL, MS, SK, and TG were responsible for the conception and design of the study and contributed to the extraction, preparation, and analysis of data. RL and MS collected and collated the data. The original draft was written by RL. RL and TG contributed to writing sections of the manuscript. All authors were involved in reviewing and editing the manuscript and agreed on the final version.

### Conflict of Interest

The authors declare that the research was conducted in the absence of any commercial or financial relationships that could be construed as a potential conflict of interest.
