# Supplementary material for: Like Father Like Son: Cultural and Genetic Contributions to Song Inheritance in an Estrildid Finch
Source: Front Psychol. 2021 Jun 4;12:654198. doi: 10.3389/fpsyg.2021.654198 (PMC8213215; doi:10.3389/fpsyg.2021.654198)
Supplement: Supplementary file 1 [file Table_1.pdf]

Supplementary Information 1

Results of mixed effect models for structural features of songs using unedited computational clusters†

Response	Social Father's phenotype	log(Age)	Pedigree	Clutch	Pedigree or Clutch
Number of notes*	0.45 p=0.0002	0.12 p=0.068	p=0.11	p=0.15	p=0.10
**Note types (manually assigned)**					
Repertoire size*	0.82 p<0.0001	0.007 p=0.88	p=0.99	p=0.98	p>0.99
Shannon entropy	0.80 p<0.0001	-0.0059 p=0.88	p=0.98	p=0.98	p>0.99
Song linearity	0.39 p=0.0006	0.0046 p=0.54	p=0.89	p=0.10	p=0.27
1st order entropy	0.81 p<0.0001	0.012 p=0.51	p=0.71	p=0.79	p=0.71
2nd order entropy	0.70 p<0.0001	0.035 p=0.056	P=0.97	p=0.94	p>0.99
**Note subtypes (computationally assigned)**					
Repertoire size*	0.61 p<0.0001	0.087 p=0.21	p>0.99	p=0.29	p=0.57
Shannon entropy	0.63 p<0.0001	0.057 p=0.39	p>0.99	p=0.39	p=0.69
Song linearity	0.23 p=0.11	-0.011 p=0.25	p=0.98	p=0.98	p>0.99
1st order entropy	0.30 p=0.017	0.051 p=0.067	p=0.84	P>0.99	p=0.96
2nd order entropy	0.10 p=0.49	0.030 p=0.13	p=0.99	P>0.99	p>0.99

*indicates response variable was log-transformed

†Across all birds, structural features were computed from a total of 676 songs with a total of 22,972 notes. For each structural feature, we studied data on 58 social father-son pairs.
