# Supplementary material for: Like Father Like Son: Cultural and Genetic Contributions to Song Inheritance in an Estrildid Finch
Source: Front Psychol. 2021 Jun 4;12:654198. doi: 10.3389/fpsyg.2021.654198 (PMC8213215; doi:10.3389/fpsyg.2021.654198)
Supplement: Supplementary file 2 [file Table_2.pdf]

Supplementary Information 2

Results of mixed-effect models for vocal consistency without one clutch that appeared influential in scatterplots†

Response	Social Father's phenotype	log(Age)	Relatedness	Clutch	Relatedness or Clutch
Note duration	0.16<br>p=0.23	-0.34<br>p=0.0022	p=0.15	p=0.30	p=0.042
Mean dominant frequency	0.34<br>p=0.033	-0.17<br>p=0.15	p>0.99	p=0.036	p=0.11
Frequency change	0.095<br>p=0.52	-0.071<br>p=0.62	p>0.99	p=0.60	p=0.87
Dynamic time warping	0.25<br>p=0.17	-0.055<br>p=0.28	p>0.99	p=0.0049	p=0.019
Spectral cross correlation (median)	0.11<br>p=0.25	-0.010<br>p=0.067	p>0.99	p=0.020	p=0.039

†Vocal consistency was computed from a total of 17,594 (note duration, mean dominant frequency); 17,011 (frequency change); or 16,786 (dynamic time warping, spectral cross correlation) notes where the same note subtype appeared multiple times in the same song. For each vocal consistency measure, we studied data on 55 social father-son pairs.
